# Early post‐treatment blood oxygenation level‐dependent responses to emotion processing associated with clinical response to pharmacological treatment in major depressive disorder

**DOI:** 10.1002/brb3.2287

**Published:** 2021-08-01

**Authors:** Rebecca J. Williams, Elliot C. Brown, Darren L. Clark, G. Bruce Pike, Rajamannar Ramasubbu

**Affiliations:** ^1^ Hotchkiss Brain Institute University of Calgary Calgary Alberta Canada; ^2^ Department of Radiology University of Calgary Calgary Alberta Canada; ^3^ Mathison Centre for Mental Health Research and Education University of Calgary Calgary Alberta Canada; ^4^ Department of Clinical Neurosciences University of Calgary Calgary Alberta Canada; ^5^ Department of Psychiatry University of Calgary Calgary Alberta Canada; ^6^ Charité‐Universitätsmedizin Berlin Corporate member of Freie Universität Berlin, Humboldt‐Universität zu Berlin, and Berlin Institute of Health, Neuroscience Research Center Berlin Germany

**Keywords:** antidepressant agents, brain mapping, emotions, facial recognition, functional magnetic resonance imaging, major depressive disorder

## Abstract

**Introduction:**

Pre‐treatment blood oxygenation level‐dependent (BOLD) functional magnetic resonance imaging (fMRI) has been used for the early identification of patients with major depressive disorder (MDD) who later respond or fail to respond to medication. However, BOLD responses early after treatment initiation may offer insight into early neural changes associated with later clinical response. The present study evaluated both pre‐treatment and early post‐treatment fMRI responses to an emotion processing task, to further our understanding of neural changes associated with a successful response to pharmacological intervention.

**Methods:**

MDD patients who responded (*n* = 22) and failed to respond (*n* = 12) after 8 weeks of treatment with either citalopram or quetiapine extended release, and healthy controls (*n* = 18) underwent two fMRI scans, baseline (pre‐treatment), and early post‐treatment (one week after treatment commencement). Participants completed an emotional face matching task at both scans.

**Results:**

Using threshold‐free cluster enhancement (TFCE) and non‐parametric permutation testing, fMRI activation maps showed that after one week of treatment, responders demonstrated increased activation in the left parietal lobule, precentral gyrus, and bilateral insula (all *P* < 0.05 threshold‐free cluster enhancement (TFCE) family‐wise error‐corrected) to negative facial expressions. Non‐responders showed some small increases in the precentral gyrus, while controls showed no differences between scans. Compared to non‐responders, responders showed some increased activation in the superior parietal lobule and middle temporal gyrus at the post‐treatment scan. There were no group differences between responders, non‐responders, and controls at baseline.

**Conclusions:**

One week after treatment commencement, BOLD signal changes in the parietal lobules, insula, and middle temporal gyrus were related to clinical response to pharmacological treatment.

## Graphical abstract

Patients diagnosed MDD who later went on to respond to medication showed robust BOLD increases after one week of treatment to an emotion processing task. There were no differences between responders, non‐responders and healthy controls at baseline, indicating that BOLD fMRI can be used to identify mediating markers of treatment response.

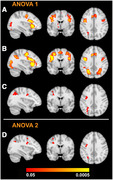

## 1 INTRODUCTION

It is important to identify which patients diagnosed with major depressive disorder (MDD) will respond to therapeutic intervention in order to expedite the challenging process of matching treatment to patient. Neuroimaging modalities have the potential to produce imaging biomarkers that indicate treatment efficacy and guide treatment selection (McGrath et al., 2013; Zarate et al., 2013). Studies utilizing functional magnetic resonance imaging (fMRI) in patients prior to treatment have shown that baseline blood oxygenation level‐dependent (BOLD) responses may be predictive of later clinical response (Dunlop et al., 2019; Mayberg et al., 1997; Phillips et al., 2015; Pizzagalli, 2011). The employment of task‐based fMRI has shown that larger pre‐treatment BOLD responses to negative emotional face stimuli, such as, faces depicting sad and fearful expressions, may be associated with symptom improvement following therapy (Chen et al., 2007; Godlewska et al., 2018; Victor et al., 2013). Pre‐treatment effective connectivity between brain regions associated with emotion processing, such as the amygdala and anterior cingulate cortex (ACC), has also shown to be indicative of later outcome to treatment with selective serotonin re‐uptake inhibitor (SSRI) escitalopram (Vai et al., 2016). Meta‐analyses of functional imaging have shown that BOLD responses in regions including the ACC, hippocampus, amygdala, and insula are very commonly implicated in MDD treatment outcomes.

Investigating early post‐treatment neural changes, in addition to baseline prediction, may provide more insight into the neural mechanisms governing clinical response to medication. There is evidence suggesting that BOLD signal changes early in treatment, within 1–2 weeks after commencing pharmacological therapy, may be associated with later clinical outcome (An et al., 2019; Davidson et al., 2003; Delaveau et al., 2016; Godlewska et al., 2016). In one study, resting‐state fMRI was utilized to investigate changes in striatal connectivity. It was found that changes in striatal functional connectivity 2 weeks after commencing treatment correlated with symptom outcome at 8 weeks (An et al., 2019). Another study characterized BOLD activation to emotional face stimuli after one week of pharmacological antidepressant treatment with escitalopram (Godlewska et al., 2016). At this post‐treatment scan, patients who showed a clinical response to medication (50% reduction in symptoms after 6 weeks of therapy) demonstrated decreases in BOLD activation to fearful faces within regions including the insula, amygdala and cingulate cortices compared to pre‐treatment baseline. The authors suggested that such results may indicate a normalization of BOLD responses after one week of antidepressant treatment (Godlewska et al., 2012), which may serve as a potential predictor of clinical response. These findings of post‐treatment BOLD signal changes within the insula, amygdala and cingulate provide novel information to complement studies using baseline data only.

Given the paucity of literature in early post‐treatment markers, further investigation into pre and early post‐treatment imaging is highly warranted. The pre‐ and post‐treatment markers represent different aspects of neurophysiology. The pre‐treatment imaging demonstrates fixed markers that are based on underlying pathophysiology, and can guide initial treatment selection. The post‐treatment neural changes are markers of response to medication which might reflect the specific effects of the pharmacological agent on the neural mechanisms associated with depression. This is why it is equally important to address medication type. Prior work investigating early post‐treatment BOLD responses (Godlewska et al., 2016) used only a single antidepressant medication, an SSRI. It would be prudent to test different medication types with different mechanisms of action when assessing brain changes following treatment, as different classes of drugs may have regional or network specific effects. In the present study, two classes of antidepressants with differential actions on serotonin transporter (5‐HTT) inhibition were implemented. Citalopram is an SSRI antidepressant (Cipriani et al., 2012) and quetiapine extended release (XR) is an atypical antipsychotic that has established efficacy as a monotherapy in randomized controlled trials of patients with MDD (Bortnick et al., 2011; Cutler et al., 2009). 5‐HTT is the major target for citalopram, and its availability in the brain predicts treatment responsiveness to SSRIs (Kugaya et al., 2004). Quetiapine has no effect on 5‐HTT, and its antidepressant effect is partly mediated through the direct inhibition of postsynaptic 5‐HT2A receptors and activation of 5‐HT1A serotonin receptors (Bauer et al., 2009).

The primary purpose of this study was to compare baseline and early post‐treatment functional BOLD responses between patients who later responded to medication; those who failed to respond, and healthy controls. BOLD responses to an emotion decision making task at pre‐treatment baseline and one‐week post‐treatment were evaluated. Clinical response was determined after 8 weeks of pharmacological treatment, with two different types of medication implemented (citalopram or quetiapine XR). Based on prior findings of post‐treatment responses in patients with MDD (An et al., 2019; Godlewska et al., 2016), it was expected that neural changes early in treatment would be found in brain regions implicated in emotion processing including the insula, amygdala and cingulate cortices, and in regions implicated in processing emotion from face expressions. Moreover, it is hypothesized that these changes would be associated with response to pharmacological treatment after 8 weeks.

## 2 MATERIALS AND METHODS

### 2.1 Participants

Data from 18 healthy controls (age = 33.1 ± 10.2 years, 8 male) and 38 patients diagnosed with MDD (36.2 ± 11.0 years, 12 male) according to DSM‐IV criteria for MDD, as assessed with the Structured Clinical Interview (First et al., 2002), were included in this study. The other inclusion criteria were: a score of (at least) 18 on the 17‐item Hamilton Rating Scale for Depression (HAM‐D) and free of psychotropic medication for a minimum of 3–4 weeks at recruitment. The exclusion criteria were as follows: 1) Any DSM‐IV Axis I disorder not defined in the inclusion criteria; 2) Major depression with mood congruent and incongruent psychotic symptoms, 3) Patients who, in the opinion of the investigator, pose an imminent risk of suicide or a danger to self or others, 4) Substance or alcohol dependence at enrolment (except dependence in full remission, and except for caffeine or nicotine dependence), as defined by DSM‐IV criteria, 5) Medical conditions that would affect absorption, distribution, metabolism, or excretion of study treatment (renal, liver, and severe gastrointestinal disorders), 6) Unstable or inadequately treated medical illness (e.g., congestive heart failure, angina pectoris, hypertension, Diabetes Mellitus) and neurological illnesses (e.g., traumatic brain injury, epilepsy, stroke, dementia, Parkinson's disease, multiple sclerosis, 7) Known intolerance or lack of response to quetiapine fumarate and citalopram as judged by the investigator, 8) Use of any of the following cytochrome P450 3A4 inhibitors in the 14 days preceding enrolment including but not limited to: Ketoconazole, itraconazole, fluconazole, erythromycin, clarithromycin, troleandomycin, indinavir, nelfinavir, ritonavir, fluvoxamine, and saquinavir, 9) Use of any of the following cytochrome P450 inducers in the 14 days preceding enrolment including but not limited to: Phenytoin, carbamazepine, barbiturates, rifampin, St. John's Wort, and glucocorticoids 10) Pregnancy or lactation, and 11) Subjects contraindicated for MRI (e.g., pregnancy, metal implants), including severe claustrophobia.

Patients were randomly assigned in a 1:1 ratio to receive treatment with either quetiapine XR fixed dose of 300 mg or citalopram 20 mg for an 8‐week period. In quetiapine group, quetiapine XR was initiated at 50 mg day^−1^ the first 2 days and 150 mg day^−1^ on day 3 and titrated to 300 mg day^−1^ by day 4. In citalopram group, citalopram was initiated at the dose of 10 mg day^−1^ and titrated to 20 mg day^−1^ by day 4. The patients in both treatment arms continued this dosage until the completion of the study protocol 8 weeks later. If patients developed side effects, the dosage of quetiapine was reduced to 150 mg day^−1^ and citalopram was reduced to 10 mg day^−1^ according to clinical judgement. Patients who could not tolerate the lowered doses were terminated from the study. Psychotherapy or any psychoactive medication including benzodiazepines was not permitted during the study. Zopiclone at the dose range of 5–7.5 mg day^−1^ were given for sedation at the discretion of the investigator. Few patients in citalopram group used Zopiclone for insomnia. Since quetiapine XR is very effective in improving insomnia, none of the patients in quetiapine group required additional sedation.

There was a total of 57 patients recruited to the study, however 19 datasets were excluded from the present analyses due to: patient inability to complete second follow‐up scans (*n* = 4), scanner‐related hardware issues resulting in lost data (*n* = 3), excessive movement/image artefact (*n* = 6), and incomplete clinical datasets due to inability to complete both scans in their entirety and data loss (*n* = 6). Data from 4 patients who did not complete the final HAM‐D were included in the comparisons of drug type in the present study, but not in comparisons of responders versus non‐responders. The mean age of all excluded patients was 37.37 ± 9.30 years, which did not differ from controls (33.06 ± 10.15 years) nor patients included in the study and classified as either a responder or non‐responder (37.85 ± 10.41 years), *P* = 0.25. One‐way analyses of variances (ANOVAs) showed that for the duration of illness since onset, there was no difference between patients excluded, and included responders and non‐responders (excluded group: *M* = 43.22 ± 64.77 months, *P* = 0.51). There were also no differences between these groups for number of episodes (excluded group: *M* = 1.94 ± 1.55, *P* = 0.07). There were 10 males/9 females excluded, and a chi‐square test showed that sex distribution did not differ between patients excluded, those included, and healthy controls (*P* = 0.46). A CONSORT flow diagram outlining the number of participants at each stage of the study is presented in the supplementary materials, in Figure S1.

After 8 weeks of treatment, patients were defined as responders if they demonstrated a ≥50% reduction in HAM‐D scores from the pre‐treatment baseline scores. This dichotomy of patients into responders and non‐responders based on 50% improvement was implemented as it was in keeping with prior literature, and therefore important for comparison and replication purposes (Godlewska et al., 2016; Vai et al., 2016; Young et al., 2020). Assessment of depression symptoms was performed using the HAM‐D at 3 time points: baseline, after one week of initiating treatment, and at 8 weeks post‐treatment. For the 34 patients grouped as responders and non‐responders in the present study, 1 had an unknown medication history. For the remaining 33, 7 (21%) were drug naïve and 26 (79%) had prior medication trials. An independent samples *t*‐test showed that responders and non‐responders did not differ in the number of previous antidepressant trials (*t*_31 _= 1.62, *P *= 0.12).

A healthy control group was included to identify illness markers at the baseline, and to control for habituation of neural response and practice effects. Control participants were screened using the Structured Clinical Interview for DSM‐IV Axis I Disorders, non‐patient version, to ensure they did not have previous or current Axis I psychiatric disorders or any family history of Axis I disorders by self‐report. HAM‐D scores ≤7 as cut off to define non‐depressed state. Control participants did not have any major unstable medical or neurological illnesses, which were determined by self‐report. This study was approved by the Calgary Conjoint Health Research Ethics Board, and informed consent was obtained from all participants prior to their participation. Data presented are part of a larger study evaluating effect of genotype on fMRI response in patients with MDD, registered at clinicaltrials.gov (NCT02132286).

### 2.2 Data acquisition

All patients completed two separate scans. The first (scan 1; pre‐treatment) was completed at baseline prior to treatment. Treatment was initiated on the same day, after the pre‐treatment scan. The second scan (scan 2; early post‐treatment) was completed 7 days after the commencement of treatment. In exceptional circumstances, due to scanner non‐availability and patient sickness, the post‐treatment scan was performed 1 or 2 days after the 7‐day trial. This happened only in 4 participants. The healthy control participants also completed two scans, one week apart without treatment. Images were collected on a 3 T GE scanner (Discovery 750, GE Healthcare Wisconsin, USA) using a 12‐channel head coil. For both scans, the same imaging protocol was utilized. This consisted of four BOLD fMRI runs (TR/TE = 2000/30 ms, 3.75 × 3.75 × 4 mm, 30 slices, 152 volumes per run) where participants simultaneously performed a behavioral task. A high‐resolution 3D T_1_‐weighted MPRAGE image (TR/TE = 9.2 ms/minimum, isotropic voxel size = 1mm^3^) was also collected.

### 2.3 Behavioral task

All participants completed the same emotional face matching task at both scans (baseline and one‐week after treatment commencement). This task has been well‐described elsewhere (Hariri et al., 2002; Ramasubbu et al., 2016). The task contained 5 experimental stimuli: Angry, fearful, happy, and sad faces, and geometrical figures. For each run, there were 60 trials (12 trials per condition). This jittered event‐related design presented each condition in a randomized order, with each stimulus presented for 3 seconds per trial. For each trial, participants were presented with a source face, and two target faces. The task involved deciding which of the two target faces matched the emotion of the source face, which the participants completed using a two‐button keypad press. Responses were recorded and non‐parametric Kruskal‐Wallis ANOVAs were performed on the reaction times and the number of correct responses. These ANOVAs determined whether there were any behavioral differences between groups (responders, non‐responders, controls) at each scan (scan 1 and scan 2) for each condition (angry, fearful, happy, sad, geometric figures). Familywise error rate was controlled using Bonferroni correction. There were 10 tests performed for reaction time and 10 for accuracy rate (i.e., a one‐way ANOVA for each of the 5 conditions at baseline and at scan 2).

### 2.4 Functional magnetic resonance imaging analyses

Preprocessing of the fMRI data was performed using SPM12 (https://www.fil.ion.ucl.ac.uk/spm/) in MATLAB and included rigid‐body linear registration for motion correction, affine registration to each subject's T_1_ image, non‐linear registration of the T_1_ image to the MNI template (Ashburner & Friston, 2005), and spatial smoothing with a 5 mm full‐width at half‐maximum Gaussian kernel. Further affine registrations were performed using Advanced Normalization Tools (Avants et al., 2011; Avants et al., 2014) between scan 1 and scan 2 images, to ensure precise registrations between these datasets. Each dataset was visually inspected for registration accuracy.

First‐level analyses modelled each of the 5 task conditions using the canonical hemodynamic response function. Only correct answers were included, and incorrect answers were modelled as effects of no interest. Six motion parameters (3 translation, 3 rotation) were also entered into the model as regressors of no interest. For all group‐level analyses, the contrast of interest was faces with negative emotional expressions (angry, fearful, and sad (AFS) faces) relative to happy expressions. This contrast of angry, fearful, sad > happy expression was chosen based on prior literature demonstrating that BOLD responses to negative emotional stimuli may be associated with later clinical outcome (Godlewska et al., 2016; Godlewska et al., 2012). The angry, fearful, sad > happy contrast images from the first‐level analyses were entered into two second‐level ANOVAs.

Two group mixed‐effects ANOVAs were performed using the Sandwich Estimator (SwE) Toolbox for Longitudinal and Repeated Measures Data in SPM12 (Guillaume et al., 2014). This toolbox was implemented because its linear mixed effects (LME) analyses account for repeated‐measures inter‐scan variance. The present study compared data from the same subjects across multiple time‐points, therefore it was essential to account for this variance rather than implement a standard random‐effects analysis (such as in SPM or FSL). For both group ANOVAs, statistical inferences were achieved using the Wild Bootstrap nonparametric permutation testing approach, and threshold‐free cluster enhancement (TFCE) (Smith & Nichols, 2009) implemented in the SwE Toolbox. These nonparametric methods were chosen to enhance signal sensitivity while controlling the Type I error rate. Non‐parametric analyses for fMRI have shown to be an ideal option for controlling false positives, compared to the standard parametric techniques implemented in the most popular fMRI analysis toolboxes (Eklund et al., 2016). TFCE is a sensitive technique for cluster‐based correction that removes the necessity of setting a minimum cluster size and height threshold, and has shown to be particularly beneficial for controlling false positives (Han et al., 2019). According to (Smith & Nichols, 2009), the TFCE approach involves calculating a TFCE value for each voxel from a statistical (e.g., T or F) map. Each voxel TFCE value is dependent on cluster height and extent, although no minimum number of contiguous voxels is set and the image is not intrinsically cluster‐thresholded. Rather, the image is transformed into voxel‐wise *P*‐values where the family‐wise error (FWE) rate can be controlled using permutation testing. In the present study, all permutation and TFCE parameters were kept consistent with the recommended settings (Guillaume et al., 2014), although the number of bootstraps was increased to 5000. Unless otherwise stated, all reported results were TFCE FWE‐corrected at *P* < 0.05.

#### 2.4.1 Analysis of variance 1

The first LME analysis was a 3 × 2 ANOVA that compared across all groups and time points. The factors were group (responders, non‐responders, controls) and time (scan 1, scan 2). Baseline HAM‐D scores were included in the model to account for any variability due to baseline depression severity. Age and sex were also included in the model. The aim of this analysis was to determine whether any groups showed differences between scans 1 and 2, and whether there were any significant differences between groups at either time point. Therefore, *F*‐tests were implemented to assess for main effects of group and scan, and their interaction. Paired comparisons were performed using one‐tailed *t*‐tests, with both directions assessed. These included within‐subject comparisons of scan (responders: Scan 1 vs. scan 2; non‐responders: scan 1 vs. scan 2; controls: scan 1 vs. scan 2), and between‐subject comparisons of group (responders; non‐responders; controls) at each time‐point (scan 1; scan 2).

#### 2.4.2 Analysis of variance 2

A second LME 2 × 2 ANOVA was performed, with this second ANOVA directly Comparing drug type groups (citalopram vs. quetiapine XR) rather than responsiveness (i.e., responders vs. non‐responders). Controls were not included in ANOVA 2, as this group did not take any pharmacological treatment. Baseline HAM‐D, age and sex were included in the model as covariates. There were four more participants in this analysis compared to ANOVA 1. These four participants corresponded to those who did not have HAM‐D scores at 8 weeks follow‐up, and could not be allocated to the responder nor non‐responder group. However, as the purpose of this second ANOVA was to characterize the effects of drug type on BOLD activation, these patients were able to be included in this analysis.

Drug type groups were compared across both scans to evaluate whether the pharmacological treatment type affected activation. Just as in ANOVA 1, *F*‐tests examined main effects and the interaction of group and scan. One‐tailed *t*‐tests (with both directions assessed) included between‐subject comparisons of each group (citalopram, quetiapine XR) at each time‐point (scan 1; scan 2) and within‐subject comparisons of time‐point (citalopram: Scan 1 vs. scan 2; quetiapine XR: scan 1 vs. scan 2).

### 2.5 Regions‐of‐interest analyses

Anatomical regions‐of‐interests (ROIs) were defined *a priori* and analyzed to compare responders, non‐responders, and controls using small‐volume correction (SVC). ROIs were chosen based on their known involvement in face and emotion processing (Fusar‐Poli, Placentino, Carletti, Allen, et al., 2009; Godlewska et al., 2016). Masks pertaining to the bilateral ACC, insula, amygdala and middle temporal gyrus (MTG) were obtained from the Harvard‐Oxford Cortical and Subcortical atlases (Desikan et al., 2006). The main effects of group and scan; the interaction of group x scan, and the paired comparisons outlined above for ANOVA 1 comparing groups and scans were run with SVC for each of the four ROIs. A small‐volume corrected threshold of *P* < 0.05 TFCE FWE‐corrected was chosen, with Bonferroni correction applied for the four ROIs for a final voxel‐wise threshold of *P* < 0.0125 TFCE FWE‐corrected.

For each participant, the BOLD percent signal changes (PSC) to negative (angry, fearful, sad) and positive (happy) valence stimuli within all ROIs were calculated. Calculations of PSC were performed using the methodology outlined by Pernet (Pernet, 2014). The beta values corresponding to the experimental condition were divided by the parameter estimates of the constant term. This constant term corresponds to the final column of the general linear model and is not user‐specified, but rather represents the implicit baseline. The actual numerator was the product of the condition beta and a scaling factor, which in this instance corresponded to the peak value of the convolved basis function in the design matrix (value of scaling factor = 0.2). Only voxels significant (*P* < 0.001 uncorrected for multiple comparisons) at the individual level were included in PSC calculations. Spearman's correlation analyses were performed between the change in BOLD PSCs between scan 2 and scan 1, and the change in HAM‐D score between baseline and 8 weeks for responders and non‐responders separately.

### 2.6 Intraclass correlation coefficients

**FIGURE 1:**
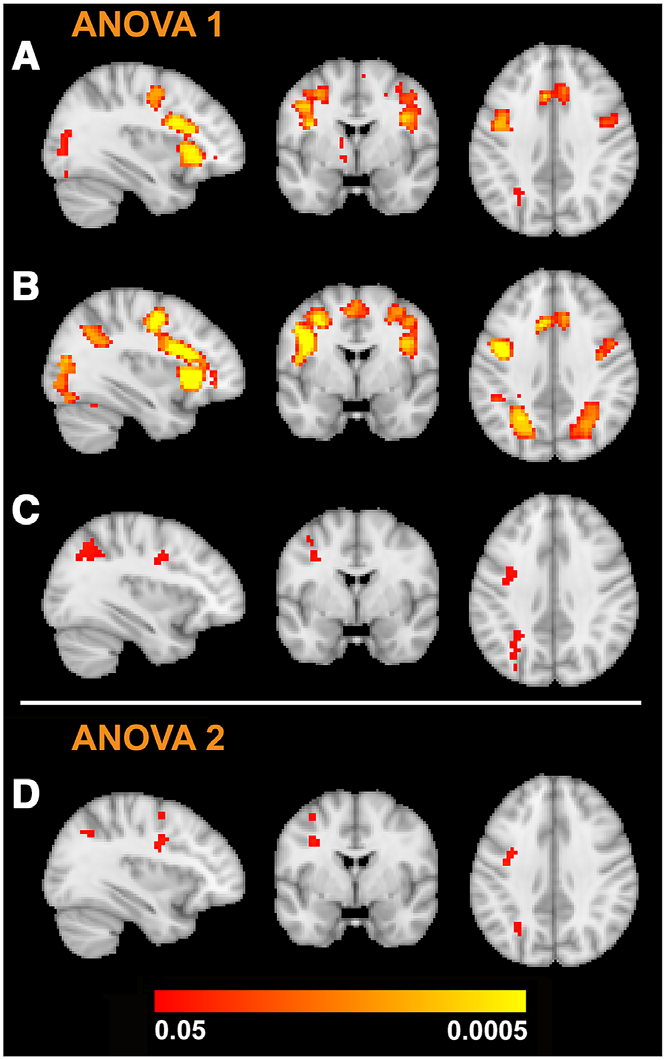
Sagittal, coronal and axial slices showing the average activation across all participants at (A) scan 1 and (B) scan 2 to negative (angry, fearful, sad) > happy facial expressions. Regions activated at both scans include the insula, inferior frontal gyrus, paracingulate gyrus, and lateral occipital cortex. Increased activation in the parietal lobules is evident at scan 2. (C) The F‐test for the group x scan interaction for analysis of variance (ANOVA) 1 resulted in activation in the left parietal lobule, angular gyrus, precentral gyrus, and inferior frontal gyrus. (D) The group x scan interaction for ANOVA 2 showed peak activation in precentral, midfrontal and lateral occipital regions. In MNI coordinates, all images are shown at level of *x* = −34 (sagittal), *y* = −1 (coronal), *z* = 36 (axial). Color bar indicates threshold‐free cluster enhancement family‐wise error‐corrected *P*‐values

To assess the stability of individual activations between the fMRI scans (scan 1 and scan 2), intraclass correlation coefficients (ICC) were calculated. ICC can be calculated for each voxel, or on a ROI basis. The ROI approach was implemented here. The ROI used was based on the significant clusters of the average group activation at scan 1 (shown in Figure 1(A)). This functional ROI was interrogated because it was expected that participants in all 3 groups (controls, responders, non‐responders) would demonstrate activation within these regions at both scans. The clusters from the group activation map were exported as a binary mask, which was used to extract the *t*‐values from these voxels in the individual activation maps, which were the *t*‐maps for the angry, fearful, sad > happy contrast, at both scans. For each participant, the mean of all *t*‐values within the ROI was used for ICC calculations. ICC were calculated using the third ICC, ICC (3,1) (Shrout & Fleiss, 1979) as implemented previously for fMRI (Caceres et al., 2009). This ICC models the two scans as fixed effects, and subjects as random effects. ICC values below 0.40 are considered poor, values between 0.40 and 0.60 are fair, and above 0.60 are good (Brandt et al., 2013).

## 3 RESULTS

### 3.1 Clinical data

Twenty‐two patients responded to medication after 8 weeks of treatment (11 citalopram, 11 quetiapine XR), and were grouped as ‘responders.’ Among these, 15 patients achieved remission (8 quetiapine XR, 7 citalopram). Twelve patients did not respond to medication (6 citalopram, 6 quetiapine XR) and were grouped as ‘non‐responders.’ Independent samples *t*‐tests showed that there were no significant differences between responders and non‐responders in baseline (1^st^) HAM‐D (*P* = 0.58), 2^nd^ HAM‐D scores (after one week of treatment) (*P* = 0.11); nor in duration of illness since onset (*P* = 0.48), duration of current episode (*P* = 0.98), and number of depressive episodes (*P* = 0.16). There was a significant difference between the two patient groups in 3^rd^ HAM‐D scores (8 weeks post‐treatment commencement; *P* < 0.0001). There was no difference between patients and controls in age (*P* = 0.24). A chi‐square test of independence showed no difference between patients and controls in sex (*P* = 0.80). Group demographic information and clinical data are shown in Table 1. The *P*‐value column in Table  displays the results of the paired comparisons tests between patients and controls (for age and sex), and responders versus non‐responders (for HAM‐D scores, illness and episode duration, and number of episodes).

**TABLE 1:** Demographic and clinical data for control and patient groups. Duration of illness since onset and duration of current episode are given in standard deviations are shown in parentheses

	**Controls (*n* = 18)**	**Responders (*n* = 22)**	**Non‐responders (*n* = 12)**	***P*‐value**
Age	33.06 (10.15)	38.00 (11.14)	37.58 (9.39)	0.24
Female (%)	10 (55.55)	14 (63.64)	8 (66.66)	0.80
1^st^ HAM‐D^†^	NA	22.00 (4.41)	21.08 (2.87)	0.58
2^nd^ HAM‐D	NA	14.59 (5.08)	17.75 (5.06)	0.11
3^rd^ HAM‐D	NA	6.18 (3.87)	14.92 (4.12)	<0.0001
Duration of illness since onset	NA	54.86 (64.86)	71.92 (70.56)	0.48
Duration of current episode	NA	34.63 (56.23)	34.09 (42.21)	0.98
Number of episodes	NA	1.73 (2.00)	4.25 (5.58)	0.16

^†^ HAM‐D = Hamilton depression scale. 1^st^ HAM‐D = baseline; 2^nd^ HAM‐D = one‐week after treatment commencement; 3^rd^ HAM‐D = 8 weeks after treatment commencement.

### 3.2 Behavioral data

For the face emotion matching task, accuracy was high across all participants, experimental conditions, and scans. For both scans, the geometrical figures condition demonstrated the highest percentage of correct responses across all participants (*M* = 99.1 ± 1.3% and *M* = 98.8 ± 3.6% at scan 1 and 2, respectively) and fearful faces was lowest in accuracy (*M* = 92 ± 7.8% and *M* = 91.4 ± 8.7% at scan 1 and 2, respectively). Reaction times were similar, with geometrical figures showing the shortest reaction times (*M* = 0.93 ± 0.14 s and *M* = 0.88 ± 0.12 s for scans 1 and 2, respectively). The one‐way ANOVAs showed that there were no significant differences between groups for accuracy rate (all *P* > 0.14) across all conditions and scans. For reaction times, fearful faces at the second scan showed the largest differences between groups (*P* = 0.02), however this was non‐significant after Bonferroni correction for multiple comparisons.

### 3.3 Functional magnetic resonance imaging analyses

#### 3.3.1 Analysis of variance 1

Averaged across all participants, robust whole‐brain activation was observed to negative faces (angry, fearful, sad) > happy faces at both scans within the bilateral insula, inferior frontal gyrus, paracingulate and lateral occipital cortex, as shown in Figure 1. Scan 1 (Figure 1(A)) showed less activation than scan 2 (Figure 1(B)), evident by smaller and fewer clusters. There were some significant voxels for the main effect of group, located in the right thalamus. The main effect of scan was non‐significant. The group x scan interaction however showed significant activation in brain regions included the left parietal lobule, precentral cortex and inferior frontal gyrus, as demonstrated in Figure 1(C). Significant clusters from the interaction are detailed in Table 2.

**TABLE 2:** All significant clusters for analysis of variance 1: Responders, non‐responders and controls at scans 1 and 2. The paired comparisons were masked so that only clusters activated in the group x scan interaction were searched. For each cluster the threshold‐free cluster enhancement‐family‐wise error corrected *P*‐value and number of voxels in the cluster are shown, with the maxima locations, test statistics and MNI coordinates (*x*, *y*, *z*)

**Contrast**	**Cluster**	***P***	***k***	**Location**	***F*/*Z***	***x***	***y***	***z***
Main effect of group								
	1	0.04	2	Thalamus	20.67	18	−7	−1
Group x scan interaction								
	1	0.03	109	Inferior parietal lobule	18.32	−30	−58	47
				Mid. occipital gyrus	17.29	−30	−70	38
				Angular gyrus	14.50	−27	−52	35
				Superior parietal lobule	10.71	−39	−40	44
	2	0.04	27	Precentral gyrus	18.82	−33	−1	35
	3	0.04	5	Inferior frontal gyrus	19.96	−39	20	29
	4	0.05	4	Mid. frontal gyrus	14.02	−39	−1	50
Responders, scan 2 > scan 1								
	1	0.0005	104	Superior parietal lobule	4.75	−30	−61	47
				Inferior parietal lobule	4.66	−36	−52	41
				Mid. occipital gyrus	4.55	−27	−67	38
				Superior parietal lobule	4.36	−30	−49	32
	2	0.004	25	Precentral gyrus	4.28	−33	−1	35
				Precentral gyrus	3.56	−39	−10	35
Non‐responders, scan 2 > scan 1								
	1	0.002	10	Precentral gyrus	3.57	−39	−7	38
	2	0.02	18	Lateral occipital cortex	2.87	−27	−64	35
Responders > non‐responders, scan 2								
	1	0.009	32	Superior parietal lobule	3.16	−30	−61	47
				Inferior parietal lobule	2.79	−36	−52	44
Responders > controls, scan 2								
	1	0.0005	95	Lateral occipital cortex	3.96	−30	−61	47
				Superior parietal lobule	3.76	−30	−52	38
				Lateral occipital cortex	3.70	−30	−79	35
	2	0.01	13	Mid. frontal gyrus	3.74	−33	−1	38
Non‐responders > controls, scan 2								
	1	0.009	17	Precentral gyrus	3.36	−36	−4	38

*k* = cluster size, *F*/*Z* = Test statistic of the maxima. F‐statistic for the main effect of group, and the group x scan interaction; Z‐statistic for within and between‐subjects contrasts.

*x*, *y*, *z* = MNI coordinates of maxima. The top maxima (up to 5) more than 10 mm apart reported.

To interrogate the significant interaction, paired comparisons were performed with SVC so that the voxel search was limited to regions activated by the interaction. For the within‐subjects comparisons, responders and non‐responders showed increased activation at scan 2 compared to scan 1. The responders showed increased activation in the left parietal lobule, while the non‐responders showed increased activation within the precentral gyrus and lateral occipital cortex. These results are detailed in Table 2. Controls showed no differences between scans. The between‐subjects comparisons indicated that the responders had increased BOLD activation compared to non‐responders at scan 2, localized to the left parietal lobule. Both responders and non‐responders showed increased activation at scan 2 compared to controls. There were no group differences at scan 1. All significant results are detailed in Table 2. The whole‐brain results for the paired comparisons were also conducted, with the results reported in the Supplementary section (Table S1 and Figure S2).

#### 3.3.2 Analysis of variance 2

There was a significant main effect of scan, with significant clusters detailed in Table 3. The main effect of group was non‐significant. There was a significant group x scan interaction for ANOVA 2, with peak activation in the precentral gyrus, lateral occipital cortex, and mid frontal gyrus. This is highlighted in Figure 1(D), and significant clusters are detailed in Table 3. For paired comparisons, search areas were limited to regions activated in the interaction. Within‐subjects comparisons showed that the patients who received citalopram showed increased activation within the precentral gyrus at scan 2 compared to scan 1. Those who received quetiapine XR showed increased in activation within the mid frontal gyrus, lateral occipital cortex and precentral gyrus at scan 2 compared to scan 1. These clusters are detailed in Table 3. Between‐subjects comparisons showed that for both scans, there no differences between drug groups. The whole‐brain results for the paired comparisons are reported in the Supplementary section (Table S2).

**TABLE 3:** Significant functional magnetic resonance imaging results for Analysis of variance 2: Citalopram and quetiapine extended release groups at scans 1 and 2. The paired comparisons were masked so that only clusters activated in the group x scan interaction were searched. For each cluster the threshold‐free cluster enhancement‐family‐wise error corrected *P*‐value and number of voxels in the cluster are shown, with maxima locations, test statistics, and MNI coordinates (*x*, *y*, *z*)

**Contrast**	**Cluster**	***P***	***k***	**Location**	***F*/*Z***	***x***	***y***	***z***
Main effect of scan								
	1	0.01	115	Precentral gyrus	23.63	−39	−10	35
				Precentral gyrus	19.11	−33	−4	56
	2	0.03	46	Mid occipital gyrus	18.65	−30	−61	35
	3	0.04	5	Mid cingulate cortex	18.62	−12	20	38
Group x scan interaction								
	1	0.03	38	Precentral gyrus	20.69	−39	−10	35
	2	0.04	7	Mid frontal gyrus	18.74	−33	−4	56
	3	0.04	25	Lateral occipital cortex	17.22	−30	−67	41
Quetiapine XR scan 2 > scan 1								
	1	0.001	7	Mid. frontal gyrus	4.37	−33	−4	56
	2	0.0005	25	Lateral occipital cortex	4.27	−33	−61	41
	3	0.0005	36	Precentral gyrus	4.07	−33	2	35
Citalopram scan 2 > scan 1								
	1	0.0005	36	Precentral gyrus	3.96	−33	−7	29

*k* = cluster size, *F*/*Z* = Test statistic of the maxima. F‐statistic for the main effect of scan and the group x scan interaction; Z‐statistic for within‐subjects contrasts.

*x*, *y*, *z* = MNI coordinates of maxima. The top maxima (up to 5) more than 10 mm apart reported.

### 3.4 Regions‐of‐interest analyses

The *a priori* anatomical ROI analyses showed a significant main effect of group for the ACC. No ROI demonstrated a significant main effect of scan nor interaction. For the paired comparisons, within‐subjects contrasts showed that the responders had increased insula activation at scan 2 > scan 1. No other ROI was significant for this contrast. The between‐subjects comparisons showed significant activation in all 4 ROIs for the responders > controls at scan 2 contrast. There was also significant activation in the MTG for responders > non‐responders contrast at scan 2. All ROI activation is detailed in Table 4.

**TABLE 4:** Significant results for *a priori* anatomical regions‐of‐interest (ROI) analyses. For each ROI, the cluster‐level threshold‐free cluster enhancement‐family‐wise error corrected *P*‐value and number of voxels in the cluster are shown, with maxima test statistics and MNI coordinates (*x*, *y*, *z*)

**Contrast**	**Cluster**	***P***	***k***	**ROI**	***F*/*Z***	***x***	***y***	***z***
Main effect of group	1	0.004	237	ACC	14.77	0	17	32
					12.37	0	23	20
					11.79	6	32	26
					10.51	6	5	29
					9.66	9	−7	41
Responders, scan 2 > scan 1								
	1	0.007	8	Insula	3.68	−30	20	−4
	2	0.007	8	Insula	3.56	33	23	2
Responders > controls, scan 2								
	1	0.001	239	ACC	4.66	−9	17	35
					4.01	6	32	26
					3.84	−3	41	17
					3.79	6	5	32
					3.39	12	14	35
	1	0.006	12	Amygdala	3.60	−27	−28	−13
					3.31	−33	−19	−13
	1	0.007	2	Insula	4.13	−36	−7	−10
	2	0.004	51	Insula	4.00	39	11	−10
					3.80	30	23	2
					3.52	39	17	2
	3	0.004	30	Insula	3.71	−36	−22	14
	4	0.005	44	Insula	3.70	−33	20	−4
	1	0.006	5	MTG	3.51	48	−25	−4
Responders > non‐responders, scan 2								
	1	0.009	2	MTG	3.82	−57	−58	11

*k* = cluster size, *F*/*Z* = Test statistic of the maxima. F‐statistic for the main effect of group; Z‐statistic for within and between‐subjects contrasts.

ACC = anterior cingulate cortex, MTG = mid temporal gyrus.

*x*, *y*, *z* = MNI coordinates of maxima. The top maxima (up to 5) more than 10 mm apart reported.

**FIGURE 2:**
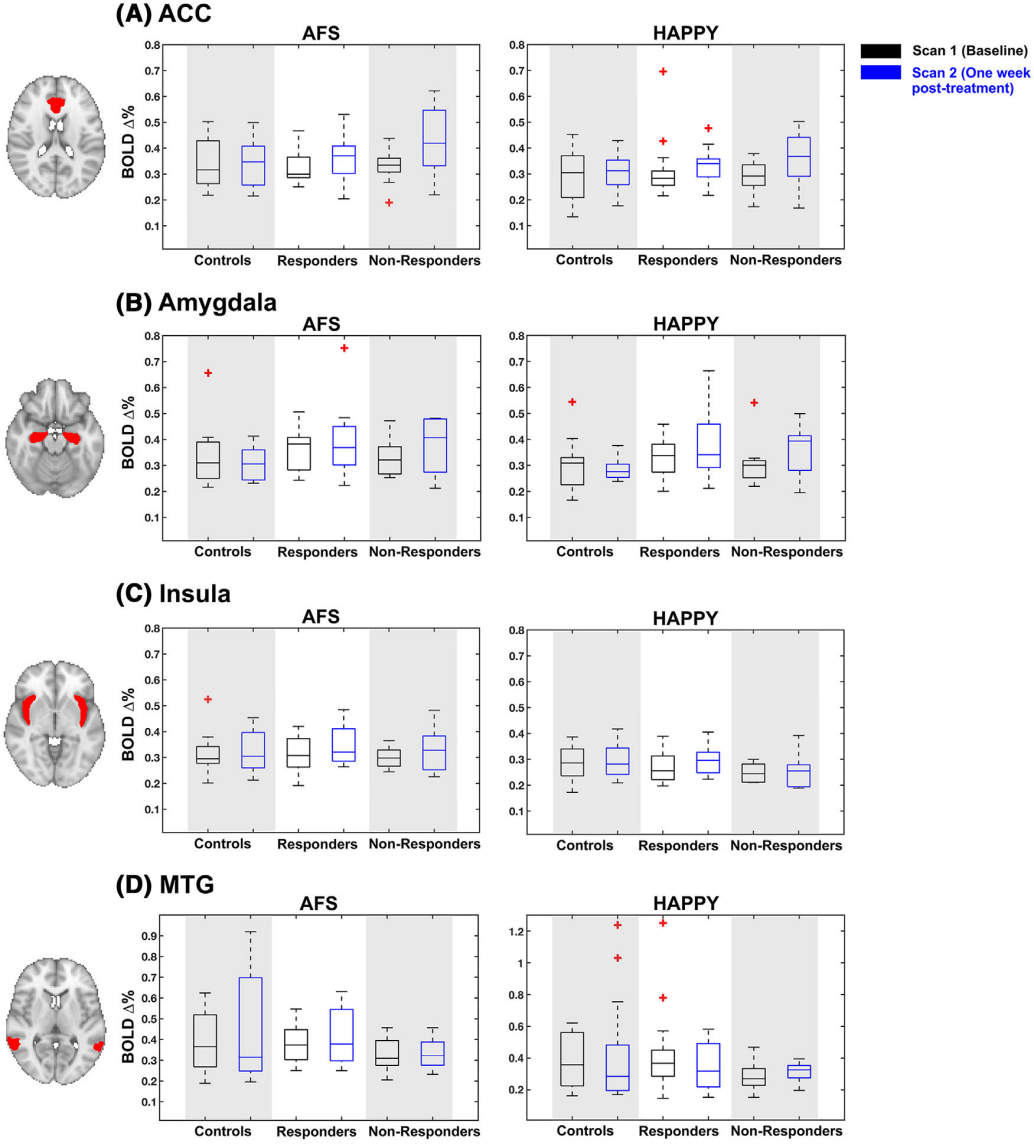
Boxplots showing blood oxygenation level‐dependent percent signal changes (PSC) for each group (controls, responders, non‐responders) within each of the four regions‐of‐interests (ROIs). The PSC to the angry, fearful, sad (AFS) faces shown in figures in the left column, and happy faces are shown in figures on the right. Both scans are shown, with scan 1 (baseline) in black boxes and scan 2 (one‐week post‐treatment) in blue. Red asterixis indicate outliers. The axial anatomical figures show on the left highlight the ROI in red, overlaid onto the MNI template image. Each row indicates the ROI: (A) Anterior cingulate cortex (ACC), (B) amygdala, (C) insula, and (C) mid temporal gyrus (MTG)

The BOLD PSC within all four ROIs for each group and scan are shown in Figure 2. The PSC to the negative faces with AFS expressions, and the PSC to the happy facial expressions, are shown. For the control group, the largest mean PSC was observed in the MTG at scan 2 to the AFS condition (*M* = 0.50 ± 0.37%). The responders group also had the largest mean PSC in the MTG, at scan 1 to the AFS condition (*M* = 0.42 ± 0.22%). The non‐responders saw the largest mean PSC in the ACC to AFS at scan 2 (*M* = 0.42 ± 0.12%). There were no significant correlations between BOLD PSC and change in HAM‐D score.

### 3.5 Intraclass correlation coefficients

**FIGURE 3:**
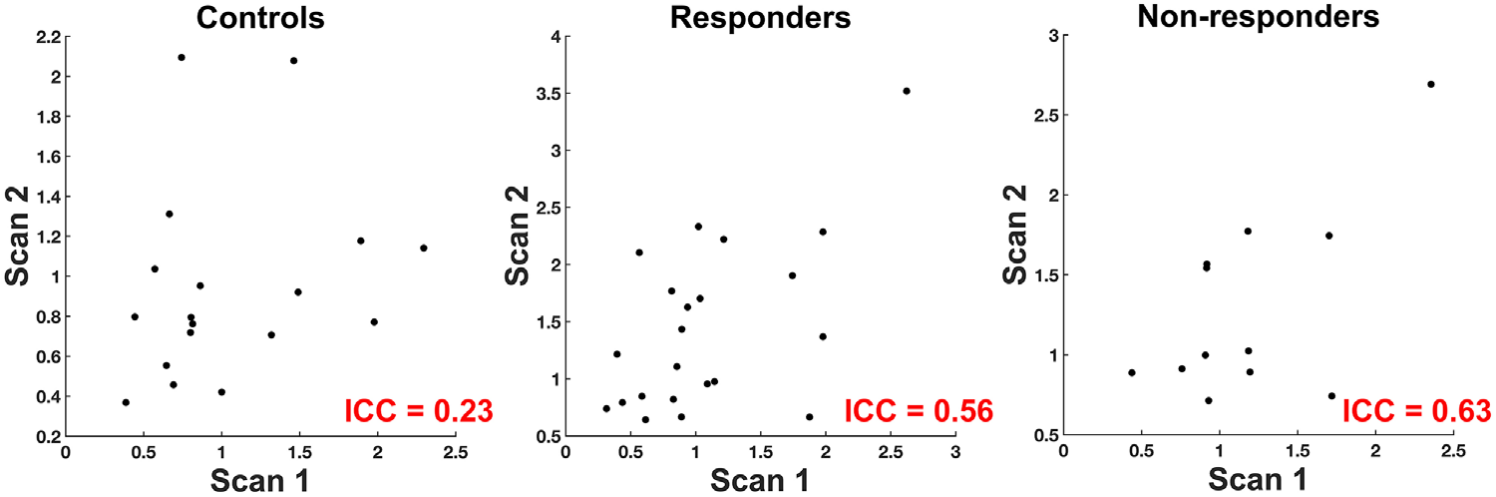
Reliability of activation between scans 1 and 2 as measured with intraclass correlation coefficients (ICC) for controls (left), responders (middle), and non‐responders (right). Axes represent mean t‐values from the functional regions‐of‐interest (ROI) used to calculate ICC. The ROI was the group activation map from scan 1 shown in Figure 1(A). Each data point represents a single subject

The non‐responders had an ICC in the good range (0.63), while for the responders it was slightly lower but still in the fair range (0.56). The controls ICC was in the poor range (0.23). Scatterplots in Figure 3 demonstrate the covarying relationship between scan 1 and scan 2 mean *t*‐values for each group. It can be seen by the axis values that responders and non‐responders had larger mean *t*‐values within the ROI than controls.

## 4 DISCUSSION

In the present study, an investigation of early fMRI changes associated with treatment response in patients with MDD was performed to identify mediating markers of clinical response to two pharmacological agents. The main finding was that patients who later responded to medication demonstrated robust increases in BOLD activation during negative emotion processing after one week of treatment. This confirmed our hypothesis that neural changes early in the treatment were related to clinical response to antidepressant treatment. We found that increased activation within the superior parietal lobule (SPL) early in the treatment was associated with improved clinical response after 8 weeks of treatment. This was contrary to expectations as the parietal lobule has not been identified as a region heavily involved in emotion processing, nor has it been routinely reported as implicated in MDD. Non‐responders showed some small increases in post‐treatment BOLD activation compared to baseline in the precentral gyrus and lateral occipital cortex, which may reflect neural changes due to the medication.

Our findings showed that task‐related BOLD signal changes early in treatment, and not pre‐treatment baseline measures, are related to later clinical response. This may suggest that mediating markers are more sensitive to clinical response than baseline markers. Godlewska et al (2016) showed results similar to our findings, indicating better group differentiation with BOLD changes early in the treatment rather than the baseline measures. However, this prior work reported decreases in BOLD responses to fearful faces in ACC, insula, and amygdala after one week of treatment in responders. On the other hand, we report BOLD increases to negative faces in the responders in these brain regions. The authors of this prior study suggested their findings of decreased activation may reflect a normalization of brain responses to stimuli with negative emotions. That is, the increased BOLD responses to negative emotions at baseline and the post‐treatment attenuation of neural responses in MDD patients might be related to pre‐treatment hyperactivity. We did not find pre‐treatment hyperactivity in these regions in MDD patients compared to controls. This lack of replication may be related to the methodological differences between the studies. This previous study used SSRI monotherapy whereas our study used two medications: Citalopram (an SSRI) and quetiapine XR. Because quetiapine XR has different mechanisms of action to SSRIs, it could be speculated that our findings reflect more general symptom recovery rather than medication‐specific neural changes. Both studies utilized emotional processing tasks, with the current study employing 5 experimental conditions (angry, fearful, happy, sad faces, and geometrical figures) while in the prior work, the authors report 2 experimental conditions (fearful and happy faces). Importantly, the previous study employed an implicit task, where subjects were required to make assessments of gender while being passively exposed to fearful and happy facial expressions. The present study employed an explicit task that requires matching faces with similar emotional expression. This task needs attention to emotion which increases cognitive demand, requiring increased recruitment of neural regions involved in cognitive control of negative emotions. Explicit emotional processing has been linked to brain regions including the temporoparietal junction, medial prefrontal cortex, inferior frontal gyrus, and medial frontal gyrus (Saxe & Powell, 2006; Siciliano & Clausi, 2020), while the implicit processing of emotions has been associated with the ACC, amygdala, insula, and postcentral gyrus (Critchley et al., 2005; Fusar‐Poli, Placentino, Carletti, Landi, et al., 2009). It is therefore possible that implicit emotional tasks recruit more limbic regions than the explicit task, which involves cortical regions for processing. However, despite these experimental differences, the present findings and the prior work of Godlewska et al. (2016) may reflect similar mechanisms of response to medication which occur shortly after treatment commencement.

A large number of studies have implemented emotion processing tasks to understand MDD and evaluate treatment efficacy, similar to the present study, as emotion processing is well‐known to be impaired in patients with MDD (Delvecchio et al., 2012; Li et al., 2018; Stuhrmann et al., 2011). Pharmacological treatment appears to improve the abnormalities in emotion regulation networks by normalizing or reversing the hypoactivity of neocortical regions and hyperactivity of limbic and paralimbic regions (subgenual cingulate region, amygdala, insula, hypothalamus) associated with depression. On the other hand, the SPL is part of the frontoparietal network which is involved in sustained attention (Ptak, 2012). While the finding reported in the present study of increased SPL activation in the responders group was not anticipated, cognitive control networks have been implicated in response prediction in patients with MDD (Crane et al., 2017). Prior work has also reported increased activation in the bilateral SPL to a facial emotion recognition task after successful, prolonged 8–12 weeks of treatment with escitalopram, an SSRI (Jiang et al., 2012). In the context of the present study, increased SPL activation in the responders group may reflect changes in attention to negative face stimuli. Patients with MDD have demonstrated attentional bias to negative stimuli, where their attention is more strongly focused on negative rather than positive stimuli (Disner et al., 2011). Brain regions implicated in attention, including the supramarginal gyrus, inferior frontal gyrus, and middle frontal gyrus, have shown reductions in activity in patients with more severe depression symptoms during an attention task (Beevers et al., 2010). It can be speculated that the increased brain activation in these regions found in the current study represent a reversal of frontoparietal reductions in these patients, although further research on this is warranted.

Previous fMRI studies investigating functional changes early into treatment as a marker of clinical response have used monotherapy, for example, escitalopram (SSRI), venlafaxine (a serotonin‐norepinephrine reuptake inhibitor, SNRI), duloxetine (SNRI), aglomelatonin (melatonin antagonist). However, our study used two types of medication (SSRI and an atypical antipsychotic) with different pharmacological profiles. Our study had the opportunity to investigate the common as well as differential neural markers of treatment response to two medication types. Given the small sample size in each treatment group, we examined the common neural markers of clinical response to both pharmacological agents by combining both treatment groups. Therefore, findings from the responders and non‐responders comparisons (ANOVA 1) reflect common mediators of clinical response to medication. However, it is important to address the possibility that the increased BOLD activity in the responders at scan 2 was heavily driven by the quetiapine XR group. The quetiapine XR group had larger BOLD activation at scan 2 compared to scan 1 in the mid frontal gyrus, lateral occipital cortex and precentral gyrus. The whole‐brain results shown in the supplementary materials (Table S2) highlight that activation was more extensive when searching beyond regions activated by the group x scan interaction, and include the inferior frontal gyrus, inferior parietal lobule, cingulate, and paracingulate cortices. The effects of quetiapine XR on frontal regions could be partially explained by norquetiapine, an active metabolite of quetiapine XR, as a norepinephrine reuptake inhibitor which increases norepinephrine and dopamine in frontal and other cortical regions. One previous study with healthy participants showed that quetiapine significantly increased resting cerebral blood flow within the prefrontal, pre and postcentral cortex, striatum and insula (Michels et al., 2016). For the citalopram group, the second ANOVA directly comparing drug types in the present study showed that one week of citalopram therapy resulted in small increases in activation in the precentral gyrus at scan 2. This finding was not significant at the whole‐brain level, as reported in the supplementary materials. Citalopram has an affinity for 5‐HTT, blocking serotonin reuptake and increasing its availability (Selvaraj et al., 2018). Most of the serotonergic cells reside in the dorsal and medial raphe nuclei of the brainstem, but innervate limbic and cortical regions (Stiedl et al., 2015). Citalopram administration has shown to attenuate amygdala BOLD responses to stimuli with negative emotions (Anderson et al., 2007; Del‐Ben et al., 2005). The responders group in the current study showed some increases in amygdala activation at scan 2, suggesting that this might have been driven by the quetiapine XR group rather than citalopram.

When addressing the study limitations, it is important to consider the lack of placebo group. The addition of a placebo group would help inform the interpretation of the results, and disentangle BOLD changes due to medication from BOLD changes due to the placebo effect (i.e., patient improvement of symptoms in the absence of an active pharmacological agent). Another consideration is the interpretation of the results. It could be argued that the increased BOLD activation found in the responders at scan 2 may represent changes due to medication, or alternately improvement of symptoms. However, the second HAM‐D collected one week after treatment commencement showed no differences between responders and non‐responders, suggesting that responders were not demonstrating significant improvement in symptoms. The small sample size of the current study is a limitation that means that the current findings should be interpreted with caution, and within the context of other research findings. Because *post hoc* power analyses are strongly discouraged (Mumford, 2012), it is difficult to assess whether null findings reported here (i.e., lack of baseline differences between groups) are due to a true lack of effect, or Type II error. Another consideration is that, for the controls, ICC was in the poor range (0.23). This is was not unexpected as prior work has reported similar ICC for task‐based fMRI (Brandt et al., 2013; Holiga et al., 2018; Plichta et al., 2012). The non‐responders showed the best ICC, which was in the good range, while for responders it was fair. These results might be explained by the patient groups demonstrating larger BOLD responses to negative faces than the controls. A final limitation that warrants consideration is the use of Zopiclone to treat insomnia. Very few participants used Zopiclone, however those who did were exclusively in the citalopram group. Zopiclone is a sedative‐hypnotic used to treat sleep disorders, and as a GABA receptor agonist, it enhances neuronal inhibition (Sanger, 2004). The influence of Zopiclone use on BOLD activation is, to the best of our knowledge, currently unknown. However, one study (Licata et al., 2011) has reported the acute effects of a similar hypnotic drug, Zolpidem, on BOLD activation. Zolpidem also increases GABA activity, and was shown to reduce BOLD activation in the occipital cortex to visual stimulation one hour following oral administration. Whether the use of Zopiclone in the current study blunted BOLD responses cannot be determined. However, ANOVA 2 showed no differences between citalopram and quetiapine XR groups, and factors such as its short duration of action and short to medium (5 h) half‐life (Terzano et al., 2003) should also be considered.

In summary, this study demonstrated that neural changes early into treatment were related to clinical response to pharmacological treatment. The identified BOLD increases in the SPL may indicate that activation of cognitive control networks may act as a mediator of treatment response. This provides some support for the hypothesis that therapeutic effect of medication may be mediated by early increases in top‐down cortical inhibition of negative emotion or negative bias. This study contributes to the emerging literature and the reliability of early post‐treatment BOLD responses as mediating markers of clinical response to medical treatments. Future research investigating early post‐treatment neural changes is highly warranted. The present study identified regions that showed early post‐treatment activation increases in responders, including the parietal lobules, insula, and MTG. It is hoped that this will lead to future studies interrogating these regions using connectivity analyses both at baseline and early post‐treatment, in order to validate these regions as implicated in MDD treatment response.

## Supporting information



Supporting Information

Supporting Information

## Data Availability

The data that support the findings of this study are available on request from the corresponding author. The data are not publicly available due to privacy or ethical restrictions.
