# Supplementary material for: Early post‐treatment blood oxygenation level‐dependent responses to emotion processing associated with clinical response to pharmacological treatment in major depressive disorder
Source: Brain Behav. 2021 Aug 1;11(8):e2287. doi: 10.1002/brb3.2287 (PMC8413787; doi:10.1002/brb3.2287)
Supplement: Supplementary file 1 — Supporting Information [file BRB3-11-e2287-s002.docx]

**Supplementary materials**

**Participants**

**Figure S1**. The CONSORT diagram showing the enrollment of participants, and the number of participants excluded at each stage of the study.

MRI and HAM-D

(n=57)

**Baseline**

## Patients diagnosed with MDD recruited to the study (n = 57)

(n = 57)

First MRI

Excluded (n=4)

- Not able to perform second scan

Allocated to Citalopram (n=29) or Quetiapine XR

(n=28)

**Allocation**

**One-week after treatment commencement**

Second MRI

MRI and HAM-D

(n=53)

Excluded (n=4)

- Not able to perform third scan and HAM-D

Third MRI

MRI and HAM-D (n=49)

**Eight-weeks after treatment commencement**

Responders (n=29)

- Citalopram (n=14)
- Quetiapine XR (n=15)

Non-responders (n=20)

- Citalopram (n=12)
- Quetiapine XR (n=8)

Excluded (n=15)

- Technical difficulties resulting in lost data (n=3)
- Excessive image artifact (n=6)
- Incomplete dataset (n=6)

Total included (n=34)

- Responders (n=22)
- Non-responders (n=12)

Analysis of first and second scans only

**Results**

*ANOVA 1*

Whole-brain within-subject comparisons revealed that non-responders and controls did not show any significant differences between scans 1 and 2. Conversely, the responders group showed extensive increases in BOLD activation at scan 2 compared to scan 1. The brain regions showing the largest increases in BOLD activation after one week of medication were the parietal lobules bilaterally. This is shown in Figure S2(A), and maxima are detailed in Table 1S.

Whole-brain between-subjects comparisons showed that responders demonstrated significantly increased BOLD activation at scan 2 compared to controls. The responders > controls comparison at scan 2 showed extensive activation with peak voxels within the cingulate cortex, inferior frontal gyrus, insula, and subcortical regions including the hippocampus and thalamus. These are shown in Figure S2(B), and clusters are described in detail in Table S1. There were very large cluster sizes for this contrast at *P* < 0.05 TFCE FWE-corrected, so Table S1 outlines the results for the cluster-level threshold of *P* < 0.005 TFCE-FWE corrected. Non-responders showed some small increases in activation compared to controls at scan 2 in the inferior frontal gyrus, Rolandic operculum and fusiform gyrus, as detailed in Table S1. No other comparison was significant.


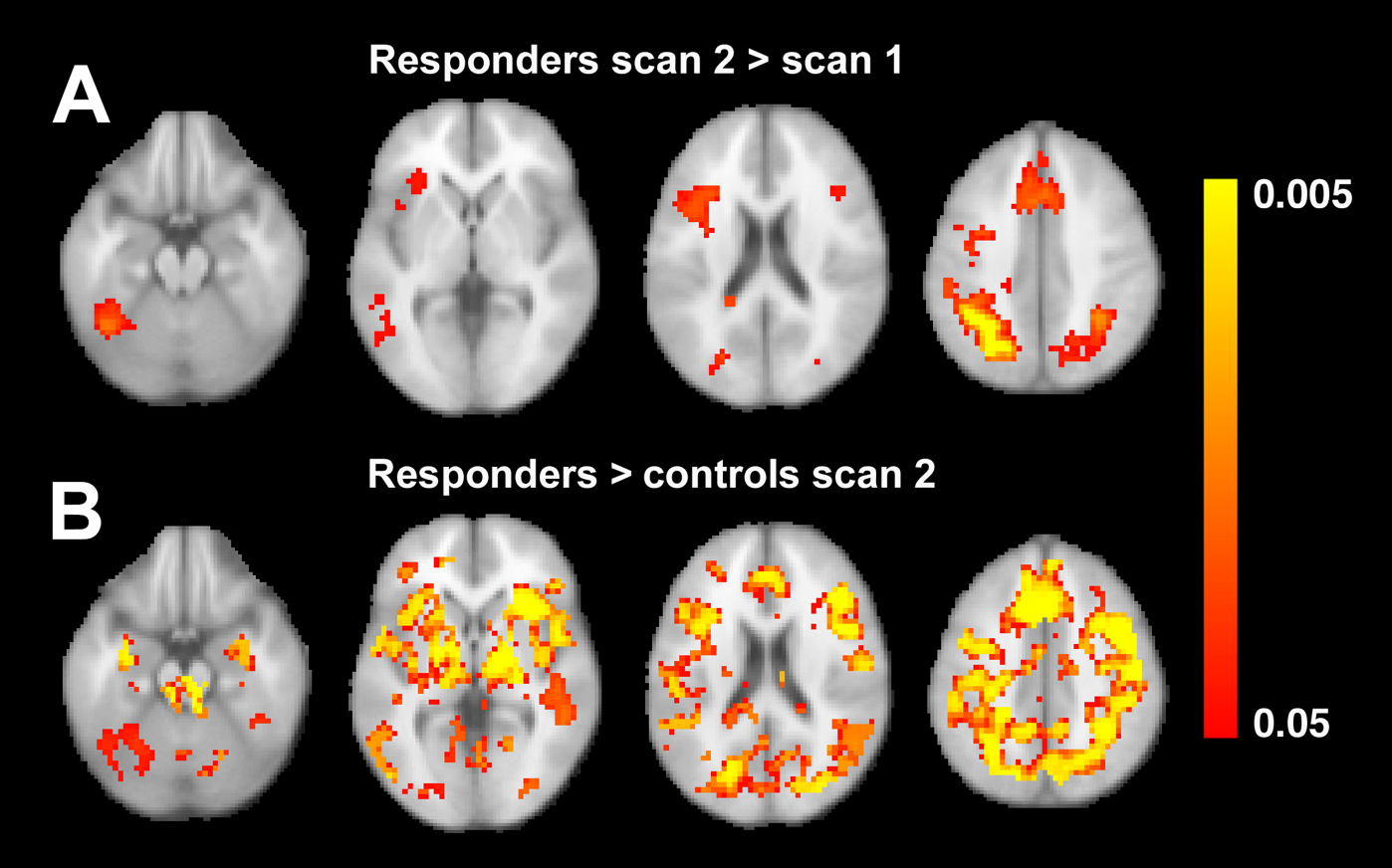


**Figure S2.** Whole-brain activation maps for angry, fearful, sad > happy faces contrast. (A) Responders showed increased activation at scan 2 compared to scan 1 within the bilateral parietal lobules, fusiform cortex and insula. (B) At scan 2, responders showed robust BOLD increases compared to controls in regions including the cingulate regions, precentral gyri, insula and thalamus. Axial figures shown at MNI coordinates (left to right) -20, 0, 20 and 40. All maps thresholded at P < 0.05 TFCE FWE-corrected. Colour bars indicate TFCE-FWE corrected P-values.

**Table S1.** All significant clusters for ANOVA 1 at the whole-brain level: responders, non-responders and controls at scans 1 and 2. For each cluster the TFCE-FWE corrected *P*-value and number of voxels in the cluster are shown, with the maxima locations, test statistics and MNI coordinates (x, y, z).

| **Contrast** | **Cluster** | ***P*** | ***k*** | **Location** | ***F/Z*** | **x** | **y** | | **z** | |
| --- | --- | --- | --- | --- | --- | --- | --- | --- | --- | --- |
| *Main effect of Group* |  |  |  |  |  |  | |  | |  |
|  | 1 | 0.04 | 2 | R. Thalamus | 20.67 | 18 | | -7 | | -1 |
| *Group x Scan Interaction* |  |  |  |  |  |  | |  | |  |
|  | 1 | 0.03 | 109 | L. Inferior parietal lobule | 18.32 | -30 | | -58 | | 47 |
|  |  |  |  | L. Mid occipital gyrus | 17.29 | -30 | | -70 | | 38 |
|  |  |  |  | L. Angular gyrus | 14.50 | -27 | | -52 | | 35 |
|  |  |  |  | L. Superior parietal lobule | 10.71 | -39 | | -40 | | 44 |
|  | 2 | 0.04 | 27 | L. Precentral gyrus | 18.82 | -33 | | -1 | | 35 |
|  | 3 | 0.04 | 5 | L. Inferior frontal gyrus | 19.96 | -39 | | 20 | | 29 |
|  | 4 | 0.05 | 4 | L. Mid frontal gyrus | 14.02 | -39 | | -1 | | 50 |
| *Responders, scan 2 > scan 1* |  |  |  |  |  |  | |  | |  |
|  | 1 | 0.004 | 1917 | L. Superior parietal lobule | 4.75 | -30 | | -61 | | 47 |
|  |  |  |  | L. Inferior parietal lobule | 4.66 | -36 | | -52 | | 41 |
|  |  |  |  | L. Mid occipital gyrus | 4.55 | -27 | | -67 | | 38 |
|  |  |  |  | L. Posterior-medial frontal | 4.19 | -6 | | 14 | | 44 |
|  |  |  |  | R. Posterior-medial frontal | 4.09 | 6 | | 17 | | 50 |
|  | 2 | 0.01 | 294 | R. Inferior parietal lobule | 5.05 | 33 | | -49 | | 41 |
|  |  |  |  | R. Superior parietal lobule | 4.59 | 30 | | -61 | | 50 |
|  |  |  |  | R. Cuneus | 3.98 | 18 | | -73 | | 38 |
|  |  |  |  | R. Mid occipital gyrus | 3.24 | 30 | | -73 | | 38 |
|  | 3 | 0.02 | 227 | L. Fusiform gyrus | 5.08 | -42 | | -58 | | -19 |
|  |  |  |  | L. Mid temporal gyrus | 3.52 | -48 | | -55 | | -4 |
|  |  |  |  | L. Cerebellum | 3.12 | -30 | | -58 | | -22 |
|  |  |  |  | L. Mid temporal gyrus | 3.07 | -48 | | -46 | | 2 |
|  | 4 | 0.03 | 127 | R. Inferior frontal gyrus | 4.28 | 42 | | 20 | | 23 |
|  | 5 | 0.04 | 47 | L. Insula | 3.68 | -30 | | 20 | | -4 |
|  | 6 | 0.05 | 16 | R. Precentral gyrus | 3.74 | 33 | | -1 | | 50 |
|  | 7 | 0.05 | 9 | R. Paracentral lobule | 3.64 | 15 | | -34 | | 53 |
| *Non-responders > controls, scan 2* |  |  |  |  |  |  | |  | |  |
|  |  |  |  |  |  |  | |  | |  |
|  | 1 | 0.04 | 12 | R. Rolandic operculum | 4.00 | 48 | | 2 | | 8 |
|  | 2 | 0.05 | 6 | R. Fusiform gyrus | 4.28 | 27 | | -85 | | -1 |
|  | 3 | 0.05 | 5 | R. Inferior frontal gyrus | 3.93 | 51 | | 11 | | 20 |
| *Responders > controls, scan 2** |  |  |  |  |  |  | |  | |  |
|  | 1 | 0.001 | 296 | L. Mid cingulate cortex | 4.66 | -9 | | 17 | | 35 |
|  |  |  |  | L. Superior medial gyrus | 4.41 | 0 | | 20 | | 41 |
|  |  |  |  | R. Anterior cingulate cortex | 4.01 | 6 | | 32 | | 26 |
|  |  |  |  | L. Superior frontal gyrus | 4.00 | -12 | | 14 | | 50 |
|  |  |  |  | R. Mid cingulate cortex | 3.62 | 6 | | 26 | | 35 |
|  | 2 | 0.002 | 265 | R. Precentral gyrus | 4.22 | 39 | | 5 | | 50 |
|  |  |  |  | R. Inferior frontal gyrus | 4.18 | 42 | | 20 | | 26 |
|  |  |  |  | R. Mid frontal gyrus | 4.09 | 42 | | 11 | | 38 |
|  | 3 | 0.003 | 66 | R. Inferior parietal lobule | 4.73 | 33 | | -49 | | 44 |
|  | 4 | 0.004 | 233 | R. Thalamus | 3.82 | 15 | | -10 | | 2 |
|  |  |  |  | R. Superior cerebellar peduncle | 3.80 | 9 | | -25 | | -7 |
|  |  |  |  | R. Internal capsule | 3.69 | 15 | | -16 | | -10 |
|  |  |  |  | L. Thalamus | 3.68 | -15 | | -4 | | 5 |
|  | 5 | 0.004 | 76 | L. Superior parietal lobule | 3.96 | -30 | | -61 | | 47 |
|  |  |  |  | L. Inferior parietal lobule | 3.76 | -30 | | -52 | | 38 |
|  | 6 | 0.004 | 39 | R. Mid cingulate cortex | 3.79 | 6 | | 5 | | 32 |
|  |  |  |  | L. Mid cingulate cortex | 3.54 | -6 | | -4 | | 35 |
|  | 7 | 0.004 | 17 | L. Insula | 4.13 | -36 | | -7 | | -10 |
|  |  |  |  | L. Hippocampus | 3.31 | -33 | | -19 | | -13 |
|  | 8 | 0.004 | 11 | L. Superior parietal lobule | 4.13 | -18 | | -73 | | 44 |
|  | 9 | 0.004 | 7 | L. Precentral gyrus | 4.27 | -45 | | 2 | | 38 |
|  | 10 | 0.004 | 10 | R. Insula | 4.00 | 39 | | 11 | | -10 |
|  | 11 | 0.004 | 61 | R. Insula | 3.95 | 27 | | 23 | | 2 |
|  | 12 | 0.004 | 43 | R. Postcentral gyrus | 3.84 | 54 | | -16 | | 41 |
|  | 13 | 0.004 | 26 | R. Posterior cingulate gyrus | 3.85 | 9 | | -19 | | 29 |
|  | 14 | 0.004 | 20 | R. Rolandic operculum | 3.86 | 48 | | 2 | | 8 |
|  | 15 | 0.005 | 51 | L. Supramarginal gyrus | 3.78 | -45 | | -28 | | 32 |
|  | 16 | 0.005 | 19 | L. Heschl’s gyrus | 3.80 | -36 | | -25 | | 14 |
|  | 17 | 0.005 | 9 | L. Anterior cingulate cortex | 3.84 | -3 | | 41 | | 17 |

*R = right, L = left, k = cluster size, F/Z =Test statistic of the maxima. F-statistic for the main effect of group and the group x scan interaction; Z-statistic for within-subjects contrast. x, y, z = MNI coordinates of maxima. The top maxima (up to 5) more than 10 mm apart reported.* **P < 0.005 TFCE-FWE corrected results shown.*

*ANOVA 2*

Whole-brain within-subjects comparisons for ANOVA 2 showed that the patients who received citalopram showed no differences between scans 1 and 2. Those who received quetiapine XR showed increases in activation at scan 2 compared to scan 1, with the largest cluster found within the left precentral gyrus and inferior frontal gyrus. Between-subjects comparisons showed that for both scans, there no differences between drug groups. All whole-brain results from ANOVA 2 are displayed in Table S2.

**Table S2.** Significant whole-brain fMRI results for ANOVA 2: citalopram and quetiapine XR groups at scans 1 and 2 (angry, fearful sad faces > happy faces contrast). For each cluster the TFCE-FWE corrected *P*-value and number of voxels in the cluster are shown, with maxima locations, test statistics and MNI coordinates (x, y, z).

| **Contrast** | **Cluster** | ***P*** | ***k*** | **Location** | ***F/Z*** | **x** | **y** | **z** |
| --- | --- | --- | --- | --- | --- | --- | --- | --- |
| *Main effect of Scan* |  |  |  |  |  |  |  |  |
|  | 1 | 0.01 | 115 | L. Precentral gyrus | 23.63 | -39 | -10 | 35 |
|  |  |  |  | L. Precentral gyrus | 19.11 | -33 | -4 | 56 |
|  | 2 | 0.03 | 46 | L. Mid occipital gyrus | 18.65 | -30 | -61 | 35 |
|  | 3 | 0.04 | 5 | L. Mid cingulate cortex | 18.62 | -12 | 20 | 38 |
| *Group x Scan Interaction* |  |  |  |  |  |  |  |  |
|  | 1 | 0.03 | 38 | L. Precentral gyrus | 20.69 | -39 | -10 | 35 |
|  | 2 | 0.04 | 7 | L. Mid frontal gyrus | 18.74 | -33 | -4 | 56 |
|  | 3 | 0.04 | 25 | L. Lateral occipital cortex | 17.22 | -30 | -67 | 41 |
| *Quetiapine XR scan 2 > scan 1* |  |  |  |  |  |  |  |  |
|  | 1 | 0.02 | 453 | L. Precentral gyrus | 4.37 | -33 | -4 | 56 |
|  |  |  |  | L. Precentral gyrus | 4.07 | -33 | 2 | 35 |
|  |  |  |  | L. Inferior frontal gyrus | 3.72 | -45 | 32 | 2 |
|  |  |  |  | L. Inferior frontal gyrus | 3.69 | -39 | 23 | 29 |
|  | 2 | 0.02 | 329 | L. Inferior parietal lobule | 4.27 | -33 | -61 | 41 |
|  |  |  |  | L. Inferior parietal lobule | 3.66 | -45 | -49 | 41 |
|  |  |  |  | L. Supramarginal gyrus | 3.58 | -33 | -46 | 32 |
|  |  |  |  | L. Lateral occipital cortex | 3.40 | -36 | -79 | 17 |
|  | 3 | 0.04 | 71 | R. Mid frontal gyrus | 4.07 | 45 | 23 | 23 |
|  |  |  |  | R. Inferior frontal gyrus | 3.48 | 39 | 11 | 23 |
|  | 4 | 0.04 | 69 | L. Posterior cingulate gyrus | 3.91 | -6 | -28 | 26 |
|  |  |  |  | R. Posterior cingulate gyrus | 3.59 | 9 | -22 | 26 |
|  |  |  |  | L. Anterior cingulate gyrus | 3.03 | -6 | -4 | 38 |
|  | 5 | 0.04 | 103 | L. Paracingulate gyrus | 3.82 | -9 | 20 | 38 |
|  |  |  |  | R. Paracingulate gyrus | 3.59 | 3 | 29 | 38 |
|  | 6 | 0.05 | 5 | L. Postcentral gyrus | 3.75 | -12 | -37 | 50 |
|  | 7 | 0.05 | 7 | R. Superior frontal gyrus | 3.75 | 3 | 20 | 59 |
|  | 8 | 0.05 | 22 | L. Insula | 3.55 | -30 | 20 | -4 |
|  | 9 | 0.05 | 17 | R. Precentral gyrus | 3.52 | 39 | -10 | 50 |

*R = right, L = left, k = cluster size, F/Z =Test statistic of the maxima. F-statistic for the main effect of scan and the group x scan interaction; Z-statistic for within-subjects contrast. x, y, z = MNI coordinates of maxima. The top maxima (up to 5) more than 10 mm apart reported.*
