# Supplementary material for: Early post‐treatment blood oxygenation level‐dependent responses to emotion processing associated with clinical response to pharmacological treatment in major depressive disorder
Source: Brain Behav. 2021 Aug 1;11(8):e2287. doi: 10.1002/brb3.2287 (PMC8413787; doi:10.1002/brb3.2287)
Supplement: Supplementary file 2 — Supporting Information [file BRB3-11-e2287-s001.pdf]

Supplementary materials

Participants

Figure S1. The CONSORT diagram showing the enrollment of participants, and the number of participants excluded at each stage of the study.

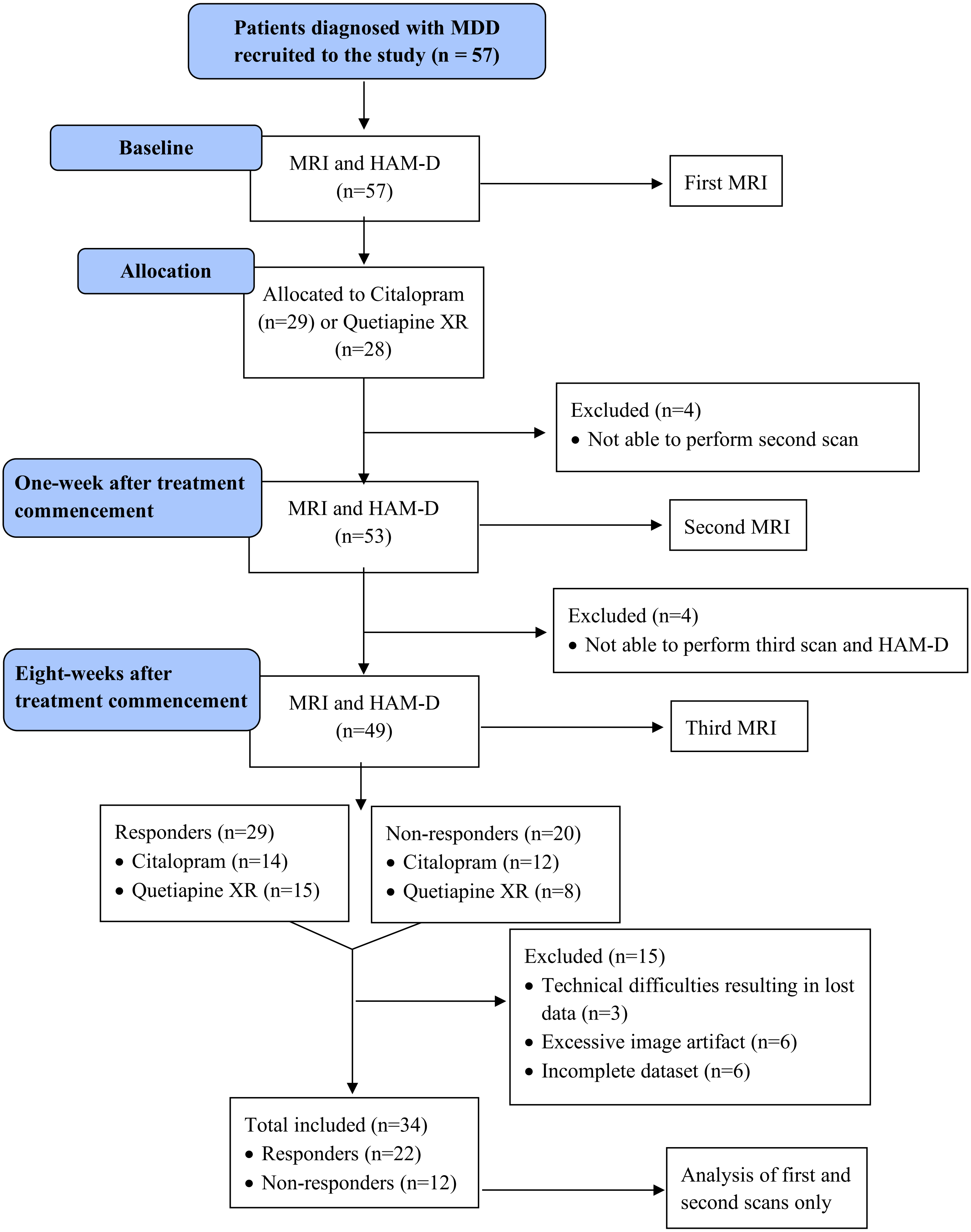
Patients diagnosed with MDD recruited to the study (n = 57)
Baseline
MRI and HAM-D (n=57)
First MRI
Allocation
Allocated to Citalopram (n=29) or Quetiapine XR (n=28)
Excluded (n=4)
• Not able to perform second scan
One-week after treatment commencement
MRI and HAM-D (n=53)
Second MRI
Excluded (n=4)
• Not able to perform third scan and HAM-D
Eight-weeks after treatment commencement
MRI and HAM-D (n=49)
Third MRI
Responders (n=29)
• Citalopram (n=14)
• Quetiapine XR (n=15)
Non-responders (n=20)
• Citalopram (n=12)
• Quetiapine XR (n=8)
Excluded (n=15)
• Technical difficulties resulting in lost data (n=3)
• Excessive image artifact (n=6)
• Incomplete dataset (n=6)
Total included (n=34)
• Responders (n=22)
• Non-responders (n=12)
Analysis of first and second scans only

Results

ANOVA 1

Whole-brain within-subject comparisons revealed that non-responders and controls did not show any significant differences between scans 1 and 2. Conversely, the responders group showed extensive increases in BOLD activation at scan 2 compared to scan 1. The brain regions showing the largest increases in BOLD activation after one week of medication were the parietal lobules bilaterally. This is shown in Figure S2(A), and maxima are detailed in Table 1S.

Whole-brain between-subjects comparisons showed that responders demonstrated significantly increased BOLD activation at scan 2 compared to controls. The responders > controls comparison at scan 2 showed extensive activation with peak voxels within the cingulate cortex, inferior frontal gyrus, insula, and subcortical regions including the hippocampus and thalamus. These are shown in Figure S2(B), and clusters are described in detail in Table S1. There were very large cluster sizes for this contrast at $P < 0.05$ TFCE FWE-corrected, so Table S1 outlines the results for the cluster-level threshold of $P < 0.005$ TFCE-FWE corrected. Non-responders showed some small increases in activation compared to controls at scan 2 in the inferior frontal gyrus, Rolandic operculum and fusiform gyrus, as detailed in Table S1. No other comparison was significant.

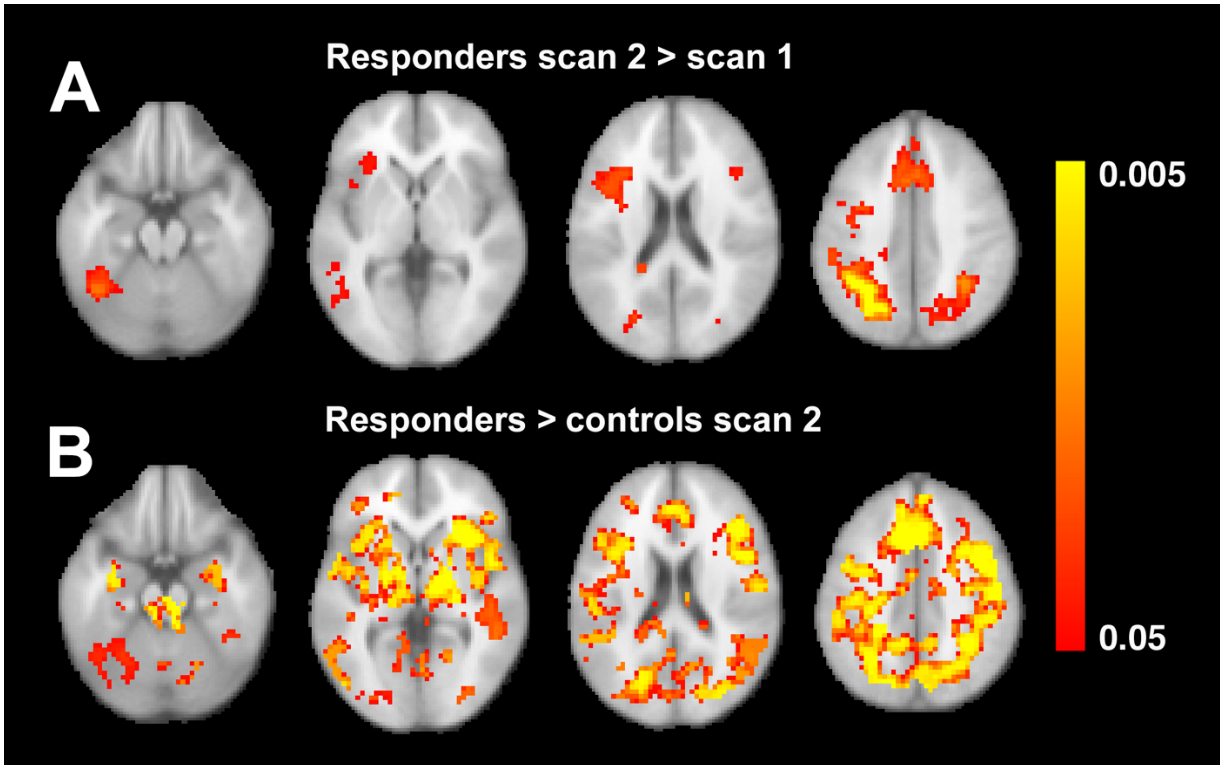

Figure S2. Whole-brain activation maps for angry, fearful, sad > happy faces contrast. (A) Responders showed increased activation at scan 2 compared to scan 1 within the bilateral parietal lobules, fusiform cortex and insula. (B) At scan 2, responders showed robust BOLD increases compared to controls in regions including the cingulate regions, precentral gyri, insula and thalamus. Axial figures shown at MNI coordinates (left to right) -20, 0, 20 and 40. All maps thresholded at $P < 0.05$ TFCE FWE-corrected. Colour bars indicate TFCE-FWE corrected *P*-values.

Table S1. All significant clusters for ANOVA 1 at the whole-brain level: responders, non-responders and controls at scans 1 and 2. For each cluster the TFCE-FWE corrected *P*-value and number of voxels in the cluster are shown, with the maxima locations, test statistics and MNI coordinates (x, y, z).

Contrast	Cluster	*P*	*k*	Location	*F/Z*	x	y	z
Main effect of Group								
	1	0.04	2	R. Thalamus	20.67	18	-7	-1
Group x Scan Interaction								
	1	0.03	109	L. Inferior parietal lobule	18.32	-30	-58	47
				L. Mid occipital gyrus	17.29	-30	-70	38
				L. Angular gyrus	14.50	-27	-52	35
				L. Superior parietal lobule	10.71	-39	-40	44
	2	0.04	27	L. Precentral gyrus	18.82	-33	-1	35
	3	0.04	5	L. Inferior frontal gyrus	19.96	-39	20	29
	4	0.05	4	L. Mid frontal gyrus	14.02	-39	-1	50
Responders, scan 2 > scan 1								
	1	0.004	1917	L. Superior parietal lobule	4.75	-30	-61	47
				L. Inferior parietal lobule	4.66	-36	-52	41
				L. Mid occipital gyrus	4.55	-27	-67	38
				L. Posterior-medial frontal	4.19	-6	14	44
				R. Posterior-medial frontal	4.09	6	17	50
	2	0.01	294	R. Inferior parietal lobule	5.05	33	-49	41
				R. Superior parietal lobule	4.59	30	-61	50
				R. Cuneus	3.98	18	-73	38
				R. Mid occipital gyrus	3.24	30	-73	38
	3	0.02	227	L. Fusiform gyrus	5.08	-42	-58	-19
				L. Mid temporal gyrus	3.52	-48	-55	-4
				L. Cerebellum	3.12	-30	-58	-22
				L. Mid temporal gyrus	3.07	-48	-46	2
	4	0.03	127	R. Inferior frontal gyrus	4.28	42	20	23
	5	0.04	47	L. Insula	3.68	-30	20	-4
	6	0.05	16	R. Precentral gyrus	3.74	33	-1	50
	7	0.05	9	R. Paracentral lobule	3.64	15	-34	53
Non-responders > controls, scan 2								
	1	0.04	12	R. Rolandic operculum	4.00	48	2	8
	2	0.05	6	R. Fusiform gyrus	4.28	27	-85	-1
	3	0.05	5	R. Inferior frontal gyrus	3.93	51	11	20
*Responders > controls, scan 2**								
	1	0.001	296	L. Mid cingulate cortex	4.66	-9	17	35
				L. Superior medial gyrus	4.41	0	20	41
				R. Anterior cingulate cortex	4.01	6	32	26
				L. Superior frontal gyrus	4.00	-12	14	50

			R. Mid cingulate cortex	3.62	6	26	35
2	0.002	265	R. Precentral gyrus	4.22	39	5	50
			R. Inferior frontal gyrus	4.18	42	20	26
			R. Mid frontal gyrus	4.09	42	11	38
3	0.003	66	R. Inferior parietal lobule	4.73	33	-49	44
4	0.004	233	R. Thalamus	3.82	15	-10	2
			R. Superior cerebellar	3.80	9	-25	-7
			R. Internal capsule	3.69	15	-16	-10
			L. Thalamus	3.68	-15	-4	5
5	0.004	76	L. Superior parietal lobule	3.96	-30	-61	47
			L. Inferior parietal lobule	3.76	-30	-52	38
6	0.004	39	R. Mid cingulate cortex	3.79	6	5	32
			L. Mid cingulate cortex	3.54	-6	-4	35
7	0.004	17	L. Insula	4.13	-36	-7	-10
			L. Hippocampus	3.31	-33	-19	-13
8	0.004	11	L. Superior parietal lobule	4.13	-18	-73	44
9	0.004	7	L. Precentral gyrus	4.27	-45	2	38
10	0.004	10	R. Insula	4.00	39	11	-10
11	0.004	61	R. Insula	3.95	27	23	2
12	0.004	43	R. Postcentral gyrus	3.84	54	-16	41
13	0.004	26	R. Posterior cingulate gyrus	3.85	9	-19	29
14	0.004	20	R. Rolandic operculum	3.86	48	2	8
15	0.005	51	L. Supramarginal gyrus	3.78	-45	-28	32
16	0.005	19	L. Heschl's gyrus	3.80	-36	-25	14
17	0.005	9	L. Anterior cingulate cortex	3.84	-3	41	17

*R = right, L = left, k = cluster size, F/Z =Test statistic of the maxima. F-statistic for the main effect of group and the group x scan interaction; Z-statistic for within-subjects contrast. x, y, z = MNI coordinates of maxima. The top maxima (up to 5) more than 10 mm apart reported. *P < 0.005 TFCE-FWE corrected results shown.*

ANOVA 2

Whole-brain within-subjects comparisons for ANOVA 2 showed that the patients who received citalopram showed no differences between scans 1 and 2. Those who received quetiapine XR showed increases in activation at scan 2 compared to scan 1, with the largest cluster found within the left precentral gyrus and inferior frontal gyrus. Between-subjects comparisons showed that for both scans, there no differences between drug groups. All whole-brain results from ANOVA 2 are displayed in Table S2.

Table S2. Significant whole-brain fMRI results for ANOVA 2: citalopram and quetiapine XR groups at scans 1 and 2 (angry, fearful sad faces > happy faces contrast). For each cluster the TFCE-FWE corrected *P*-value and number of voxels in the cluster are shown, with maxima locations, test statistics and MNI coordinates (x, y, z).

Contrast	Cluster	*P*	*k*	Location	*F/Z*	x	y	z
Main effect of Scan								
	1	0.01	115	L. Precentral gyrus	23.63	-39	-10	35
				L. Precentral gyrus	19.11	-33	-4	56
	2	0.03	46	L. Mid occipital gyrus	18.65	-30	-61	35
	3	0.04	5	L. Mid cingulate cortex	18.62	-12	20	38
Group x Scan Interaction								
	1	0.03	38	L. Precentral gyrus	20.69	-39	-10	35
	2	0.04	7	L. Mid frontal gyrus	18.74	-33	-4	56
	3	0.04	25	L. Lateral occipital cortex	17.22	-30	-67	41
Quetiapine XR scan 2 > scan 1								
	1	0.02	453	L. Precentral gyrus	4.37	-33	-4	56
				L. Precentral gyrus	4.07	-33	2	35
				L. Inferior frontal gyrus	3.72	-45	32	2
				L. Inferior frontal gyrus	3.69	-39	23	29
	2	0.02	329	L. Inferior parietal lobule	4.27	-33	-61	41
				L. Inferior parietal lobule	3.66	-45	-49	41
				L. Supramarginal gyrus	3.58	-33	-46	32
				L. Lateral occipital cortex	3.40	-36	-79	17
	3	0.04	71	R. Mid frontal gyrus	4.07	45	23	23
				R. Inferior frontal gyrus	3.48	39	11	23
	4	0.04	69	L. Posterior cingulate gyrus	3.91	-6	-28	26
				R. Posterior cingulate gyrus	3.59	9	-22	26
				L. Anterior cingulate gyrus	3.03	-6	-4	38
	5	0.04	103	L. Paracingulate gyrus	3.82	-9	20	38
				R. Paracingulate gyrus	3.59	3	29	38
	6	0.05	5	L. Postcentral gyrus	3.75	-12	-37	50
	7	0.05	7	R. Superior frontal gyrus	3.75	3	20	59
	8	0.05	22	L. Insula	3.55	-30	20	-4
	9	0.05	17	R. Precentral gyrus	3.52	39	-10	50

R = right, L = left, k = cluster size, F/Z =Test statistic of the maxima. F-statistic for the main effect of scan and the group x scan interaction; Z-statistic for within-subjects contrast. x, y, z = MNI coordinates of maxima. The top maxima (up to 5) more than 10 mm apart reported.
